# Differentiation between various types and subtypes of intracranial meningiomas with advanced MRI

**DOI:** 10.4102/sajr.v26i1.2480

**Published:** 2022-10-26

**Authors:** Mousam Panigrahi, Narendra K. Bodhey, Saroj K. Pati, Nighat Hussain, Anil K. Sharma, Arvind K. Shukla

**Affiliations:** 1 Department of Radiodiagnosis, All India Institute of Medical Sciences, Raipur, India; 2 Department of Pathology and Laboratory Medicine, All India Institute of Medical Sciences, Raipur, India; 3 Department of Neurosurgery, All India Institute of Medical Sciences, Raipur, India; 4 Department of Community and Family Medicine, All India Institute of Medical Sciences, Raipur, India

**Keywords:** meningioma, ASL, T1 perfusion, ADC, DTI

## Abstract

**Background:**

Meningiomas are the most prevalent of all intracranial tumours. Although they are mostly benign, about 20% of meningiomas are atypical or malignant. Knowledge of their histologic grade can be clinically useful while planning surgery.

**Objectives:**

To differentiate between various grades and subtypes of meningiomas with advanced MR parameters.

**Method:**

We assessed the advanced MR imaging characteristics of 27 histopathologically confirmed meningiomas on a 3T MRI, of which 23 were grade I meningiomas (2 fibroblastic, 9 meningothelial, 9 transitional, 3 unspecified) and 4 were grade II/III meningiomas (2 atypical, 1 papillary, 1 anaplastic). Analysis of the ADC, FA, λ1, λ2, λ3 and mean diffusivity was performed using standard post-processing software.

**Results:**

The mean size of atypical meningiomas (5.9 cm ± 0.7 cm) was significantly higher (*p* = 0.038, 95% confidence interval [CI]) than that of typical meningiomas (4.6 cm ± 1.6 cm) with a cut-off value of 6.05 cm (75% sensitivity and 87% specificity). The mean cerebral blood flow (CBF) (ASL) of atypical meningiomas (286.70 ± 8.06) was significantly higher (*p* = 0.0000141, 95% CI) than that of typical meningiomas (161.09 ± 87.04) with a cut-off value of 276.75 (66.7% sensitivity and 75% specificity). Among the typical meningiomas, transitional subtypes had the lowest ADC. High FA and planar coefficient (CP) values and low λ3 and spherical coefficient (CS) values were seen in fibroblastic meningiomas. Fibroblastic meningiomas also showed the lowest vascularity among typical meningiomas.

**Conclusion:**

Tumour size and ASL perfusion are two parameters that could differentiate between typical and atypical meningiomas while ADC, FA, λ3, CP, CS, rCBF and rCBV may be helpful in distinguishing different subtypes of typical meningiomas.

## Introduction

Meningiomas are the most prevalent of all intracranial tumours, constituting 30% of all primary intracranial neoplasms.^1^ They appear in middle-aged individuals and are derived from arachnoid cap cells.^2,3^

Although they are mostly benign, up to 20% of meningiomas are atypical or malignant and are more aggressive with higher recurrence rates.^4,5^ Initial tumour resection extent and histologic grade are important predictors for tumour recurrence.^6^ Therefore, knowledge of histologic grade can be clinically useful while planning surgery and adjunctive radiation therapy.

Because of intricate microstructural barriers in brain tissue, such as white matter tracts, cell membranes and capillary vessels, water molecules prefer to diffuse with direction (anisotropic diffusion) rather than uniformly in all directions (isotropic diffusion).^7^ Diffusion tensor imaging (DTI) offers data on the magnitude and directionality of water diffusion.^8^ When compared with classic or typical meningiomas, atypical meningiomas show higher diffusion anisotropy (fractional anisotropy values).^7^

In their literature review, the authors were unable to document any manuscripts comparing meningioma characteristics based on the combination of DWI (diffusion-weighted imaging), DTI and perfusion, along with histopathological correlation. This research therefore sought to distinguish between the various grades and subtypes of meningiomas using advanced MR parameters.

## Materials and Methods

This prospective, observational institution-based study was conducted over 22 months from 03 March 2019 to 31 December 2020. A total of 47 patients with a clinico-radiological diagnosis of meningiomas underwent MRI with conventional and advanced MR sequences. Of these, 12 patients did not undergo surgery for various reasons and no histopathological confirmation was available. Of the remaining 35 patients, eight underwent surgery but were not meningiomas on HPE (histopathological examination). Hence these eight were also excluded from the analysis. Thus, we assessed the MR imaging characteristics of 27 histopathologically confirmed cases of meningiomas.

### Brain MR Image Acquisition

Images were acquired on a Discovery MR750w GEM – 70 cm – 3.0 T MRI scanner, GE, Chicago, Illinois, United States using a 16-channel bird cage coil. The standardised Brain MR protocol included axial T1, axial T2, axial FLAIR, coronal T2, sagittal T1, sagittal SPGR, axial DWI (b0 and b2500 s/mm^2^), axial SWI, axial DTI, axial 3D arterial spin labelling (ASL). This was followed by injection of intravenous contrast (0.1 mL/kg of gadopentetate dimeglumine [Magnevist], Schering, Berlin, Germany) with immediate dynamic susceptibility contrast (DSC) MR perfusion imaging, post-contrast T1 in the axial, coronal and sagittal planes. MR spectroscopy was also performed.

### Post-Processing

Standard post-processing software (Ready View) was used for image analysis. Circular regions of interest (ROIs) with areas ranging from 25 mm^2^ to 50 mm^2^ were placed centrally within the solid-enhancing area of all meningiomas and peritumoural area if oedema was present. Regions of interest were then automatically transferred to the apparent diffusion coefficient (ADC), fractional anisotropy (FA), mean diffusivity (MD), eigen values in 3 orthogonal directions (λ1, λ2 & λ3), relative cerebral blood flow (rCBF), relative cerebral blood volume (rCBV), mean transit time (MTT) and cerebral blood flow (CBF) using arterial spin labelling (ASL) maps. Regions of interest were copied onto the corresponding contralateral normal-appearing white matter (NAWM) in each patient to obtain the ADC, FA, MD, λ1, λ2 & λ3, rCBF, rCBV, MTT and CBF (ASL) values for the purpose of normalisation. Linear coefficient (CL), planar coefficient (CP) and spherical coefficient (CS) values were calculated using the following algorithms:

CL=(λ1−λ2)/(λ1+λ2+λ3) [Eqn 1]

CP=2.(λ2−λ3)/(λ1+λ2+λ3) [Eqn 2]

CS=3.λ3/(λ1−λ2+λ3) [Eqn 3]

MR spectroscopy was interpreted using the Ready View software and the metabolite peaks in different areas were recorded.

### Statistical Analysis

The largest dimension of each meningioma was recorded. Mean ADC, FA, MD, λ1, λ2 & λ3, rCBF, rCBV, MTT and CBF (ASL) values were recorded in the lesion, peritumoural oedema and in the contralateral NAWM. Comparisons between solid-enhancing areas of typical and atypical meningiomas and corresponding contralateral NAWM were performed with use of paired *t* tests. Mean absolute values of ADC, FA, MD, λ1, λ2 & λ3, rCBF, rCBV, MTT and CBF (ASL) of solid-enhancing areas and peritumoural oedema, as well as the distribution of tensor shapes of the solid-enhancing areas for the two tumour types were compared with the independent sample *t* test (two-tailed; unequal variance). Microsoft Excel and IBM Statistical Package for Social Sciences (SPSS) Statistics version 26 software were used for statistical analysis, and *p*-values less than 0.05 were considered to indicate statistically significant differences. Cut-off values were estimated using ROC (receiver operator characteristic) curve analysis, where the differences were statistically significant.

### Ethical considerations

An application for full ethical approval was made to the Institute Ethics Committee and ethics consent was received on 02 March 2019. The ethics approval number is AIIMSRPR/IEC/2019/246. All procedures performed in the study were in accordance with the ethical standards of the institutional ethics committee and with the 1964 Helsinki Declaration and its later amendments. Written informed consent was requested from subjects who were willing to participate in the study.

## Results

**FIGURE 1:**
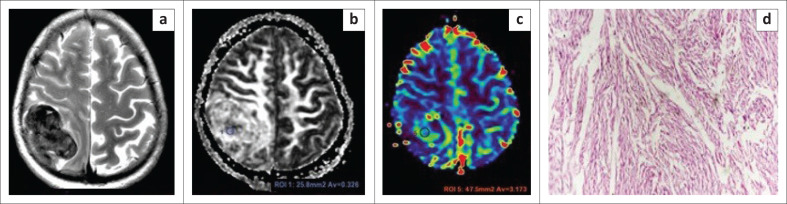
Images of a 40-year-old female who presented with headache. Axial T2WI (a) demonstrates the typical hypointense appearance of a fibroblastic meningioma. The lesion reveals a high fractional anisotropy value (b), and lower relative cerebral blood volume (c) value. The patient underwent surgery and histopathological photomicrograph (d) indicated tumour comprised of sheets of spindle cells with indistinct cell boundaries (Hematoxylin and eosin staining, ×10) confirming a fibroblastic meningioma.

**FIGURE 2:**
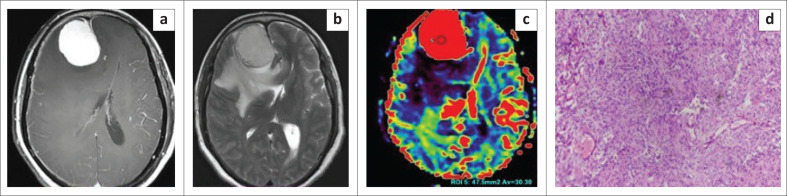
Images of a 42-year-old female who presented with headache. A well-defined globular extra-axial lesion is noted in the anterior right parafalcine location. The lesion shows homogeneous avid post-contrast enhancement (a), which is a typical feature of meningothelial meningioma. An enhancing dural tail is also demonstrated. The lesion appears homogeneously hyperintense on axial T2WI (b). Adjacent brain oedema is seen appearing hyperintense on T2WI. The lesion shows high relative cerebral blood volume values (c). The patient underwent surgery and histopathological photomicrograph (d) illustrated tumour comprised of syncytia of epithelial cells with indistinct cell boundaries (Hematoxylin and eosin staining, ×10) confirming the meningothelial subtype of meningioma.

**FIGURE 3:**
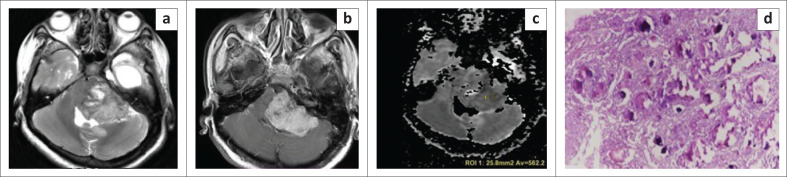
Images of a 20-year-old female who presented with bilateral hearing loss. A well-defined lobulated extra-axial lesion is seen in the left cerebellopontine angle region. The lesion is heterogeneously hyperintense on axial T2WI (a). It is causing mass effect on the 4th ventricle with resultant upstream dilatation of ventricular system. The dilated temporal horn of left lateral ventricle is seen on axial T2WI (a). The lesion shows heterogeneous post-contrast enhancement (b) which is a typical feature of transitional meningioma. Low apparent diffusion coefficient values (c) are seen. The patient underwent surgery and histopathological photomicrograph (d) illustrated tumour comprised of syncytia of epithelial cells with prominent psammoma bodies confirming the transitional subtype of meningioma (Hematoxylin and eosin staining, ×10).

**FIGURE 4:**
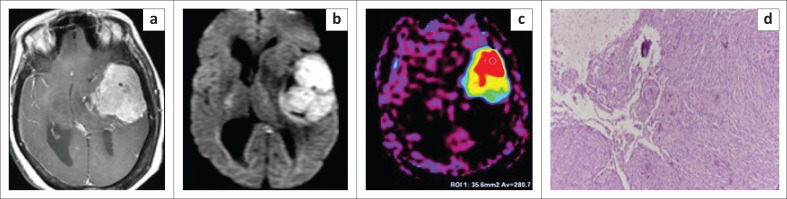
Images of a 62-year-old female who presented with dysphasia and difficulty in walking. A relatively well-defined lobulated extra-axial lesion was seen along the left temporal lobe. It revealed heterogeneous post-contrast enhancement (a). Brain-tumour interface was indistinct at a few sites. The lesion demonstrated diffusion restriction on axial diffusion-weighted imaging - apparent diffusion coefficient (ADC) not included (b) and a high cerebral blood flow value on arterial spin labelling (c). After surgery, histopathological photomicrograph (d) revealed tumour comprised of syncytia of epithelial cells with brain invasion (Hematoxylin and eosin staining, ×10) confirming the diagnosis of atypical meningioma.

Of the 27 cases, 23 (85.2%) were typical meningiomas (Grade I) and four (14.8%) were atypical meningiomas (Grades II or III). Among the 23 typical meningiomas, two were fibroblastic, nine were meningothelial, nine were transitional and three were unspecified types. Among the atypical group, one was anaplastic, one was papillary and two were atypical meningiomas. The representative cases are demonstrated in Figure 1, Figure 2, Figure 3 and Figure 4.

### Typical versus Atypical Meningiomas

Comparison between typical and atypical meningiomas based on various parameters is displayed in Table 1.

**TABLE 1:** Typical versus atypical meningiomas.

Parameters	Typical meningiomas (*n* = 23)	Atypical meningiomas (*n* = 4)	*p*
Age (years)	46.4 ± 12.8	42.8 ± 14.8	0.67
Tumour size (cm)	4.6 ± 1.6	5.9 ± 0.7†	0.038*
		7.6 ± 3.4	0.17
ADC (× 10^-3^ mm^2^/s)	0.68 ± 0.11	0.57 ± 0.12	0.16
FA	0.23 ± 0.08	0.26 ± 0.09	0.55
MD (× 10^-3^ mm^2^/s)	0.88 ± 0.14	0.86 ± 0.26	0.93
λ1 (× 10^-3^ mm^2^/s)	1.08 ± 0.13	1.10 ± 0.18	0.85
λ2 (× 10^-3^ mm^2^/s)	0.88 ± 0.14	0.85 ± 0.22	0.83
λ3 (× 10^-3^ mm^2^/s)	0.71 ± 0.16	0.68 ± 0.19	0.79
CL (%)	7.68 ± 2.91	9.99 ± 5.81	0.49
CP (%)	13.01 ± 8.71	13.13 ± 6.15	0.98
CS (%)	79.31 ± 9.96	76.89 ± 10.81	0.70
rCBF (mL/100 mg/min)	78.81 ± 72.37	66.37 ± 46.72	0.67
rCBV (mL/100 mg)	13.38 ± 10.68	9.97 ± 6.68	0.43
MTT (s)	10.55 ± 2.81	9.92 ± 1.86	0.59
CBF (ASL) (mL/100 mg/min)	161.09 ± 87.04	286.7 ± 8.06	0.0000141*

FA, fractional anisotropy; MD, mean diffusivity ; CL, linear coefficient; CP, planar coefficient; CS, spherical coefficient; rCBF, relative cerebral blood flow; rCBV, relative cerebral blood volume; MTT, mean transit time; CBF, cerebral blood flow; ASL, arterial spin labelling; ACD, apparent diffusion coefficient.

* , These are the significant *p*-values < 0.05.

† , This mean is after excluding a single outlier with a size of 12.7 cm.

Tumour size and CBF (ASL) were the two parameters that showed significant differences between the two groups. The longest dimension of each meningioma was recorded for analysis of their sizes. Typical meningiomas had sizes ranging from 2.2 cm to 8.7 cm with a mean of 4.6 cm. Atypical meningiomas had sizes between 5.2 cm and 12.7 cm with a mean size of 5.9 cm. The mean size of atypical meningiomas was significantly higher than that of typical meningiomas (*p* = 0.038, 95% confidence interval [CI]). Using ROC curve analysis, a size cut-off of 6.05 cm was determined to differentiate between the two entities with 75.0% sensitivity and 87% specificity.

The differences in the mean CBF values of typical and atypical meningiomas were also statistically significant (*p* ≤ 0.05). Receiver operator characteristic curve analysis yielded a cut-off value of 276.75, which could differentiate between typical and atypical meningiomas with 66.7% sensitivity and 75.0% specificity.

There were no discernible differences between the two groups for any of the other criteria included in Table 1.

### Subtypes of Typical Meningiomas

Comparison between the different subtypes of typical meningiomas based on various parameters is displayed in Table 2, and their group wise comparison for *p*-values and cut-off values is displayed in Table 3.

**TABLE 2:** Subtypes of typical meningiomas.

Parameters	Fibroblastic (*F*) (*n* = 2)	Meningothelial (*M*) (*n* = 9)	Transitional (*T*) (*n* = 9)
Age (years)	49.0 ± 12.7	53.4 ± 7.9	42.0 ± 15.7
Tumour size (cm)	5.4 ± 0.7	3.7 ± 1.2	4.8 ± 1.5
ADC (× 10^-3^ mm^2^/s)	0.76 ± 0.15	0.73 ± 0.09	0.66 ± 0.07
FA	0.30 ± 0.02	0.20 ± 0.07	0.22 ± 0.07
MD (× 10^-3^ mm^2^/s)	0.88 ± 0.13	0.90 ± 0.14	0.88 ± 0.15
λ1 (× 10^-3^ mm^2^/s)	1.13 ± 0.03	1.09 ± 0.15	1.09 ± 0.13
λ2 (× 10^-3^ mm^2^/s)	0.90 ± 0.02	0.90 ± 0.14	0.89 ± 0.15
λ3 (× 10^-3^ mm^2^/s)	0.60 ± 0.03	0.76 ± 0.13	0.73 ± 0.18
CL (%)	8.84 ± 0.44	7.06 ± 3.09	7.60 ± 3.25
CP (%)	22.61 ± 2.01	10.12 ± 7.23	12.31 ± 8.90
CS (%)	68.55 ± 1.57	82.81 ± 6.80	80.08 ± 10.96
rCBF (mL/100 mg/min)	38.65 ± 14.84	91.63 ± 52.20	89.15 ± 100.28
rCBV (mL/100 mg)	5.49 ± 3.15	17.81 ± 10.10	12.94 ± 12.24
MTT (sec)	9.12 ± 0.44	11.03 ± 3.52	9.87 ± 2.35
CBF (ASL) (mL/100 mg/min)	99.11	199.53 ± 113.72	224.02 ± 169.57

Note: Data are presented in mean ± s.d.

s.d., standard deviation; FA, fractional anisotropy; MD, mean diffusivity; CL, linear coefficient; CP, planar coefficient; CS, spherical coefficient; rCBF, relative cerebral blood flow; rCBV, relative cerebral blood volume; MTT, mean transit time; CBF, cerebral blood flow; ASL, arterial spin labelling; ADC, apparent diffusion coefficient.

**TABLE 3:** Comparison between different subtypes of typical meningiomas.

Parameters	*F* vs *M*	*F* vs *T*	*M* vs *T*
Age	0.70	0.58	0.07
Size	0.09	0.44	0.13
ADC	0.83	0.46	**0.005**
	-	-	**0.667**
	-	-	**Sn: 88.9%**
	-	-	**Sp: 77.8%**
FA	**0.01**	**0.038**	0.46
	**0.282**	**0.286**	-
	**Sn: 100.0%**	**Sn: 100.0%**	-
	**Sp: 100.0%**	**Sp: 77.8%**	-
MD	0.87	0.98	0.80
λ1	0.43	0.39	0.99
λ2	0.92	0.82	0.92
λ3	**0.014**	0.09	0.69
	**0.64**	-	-
	**Sn: 88.9%**	-	-
	**Sp: 100.0%**	-	-
CL	0.13	0.30	0.72
CP	**0.0025**	**0.013**	0.57
	**16.52**	**20.02**	-
	**Sn: 100.0%**	**Sn: 100.0%**	-
	**Sp: 88.9%**	**Sp: 88.9%**	-
CS	**0.0004**	**0.01**	0.53
	**73.79**	**73.79**	-
	**Sn: 88.9%**	**Sn: 88.9%**	-
	**Sp: 100.0%**	**Sp: 100.0%**	-
rCBF	**0.034**	0.18	0.94
	**59.83**	-	-
	**Sn: 66.7%**	-	-
	**Sp: 100.0%**	-	-
rCBV	**0.02**	0.14	0.37
	**7.82**	-	-
	**Sn: 88.9%**	-	-
	**Sp: 100.0%**	-	-
MTT	0.15	0.39	0.42
CBF (ASL)	-	-	0.73

Note: *P*-values; cut-off values with sensitivity (Sn) and specificity (Sp). Values in bold indicate statistically significant difference *p* < 0.05, with a cut off value in the next row and the sensitivity and specificity for that cut off.

FA, fractional anisotropy; MD, mean diffusivity; CL, linear coefficient; CP, planar coefficient; CS, spherical coefficient; rCBF, relative cerebral blood flow; rCBV, relative cerebral blood volume; MTT, mean transit time; CBF, cerebral blood flow; ASL, arterial spin labelling; ACD, apparent diffusion coefficient; F, fibroblastic; M, meningothelial; T, transitional.

### MR Spectroscopy

Of the 27 patients, MR spectroscopy yielded analysable results in 22 patients. Among these 22 patients, 20 were typical meningiomas and two were atypical meningiomas. Nineteen of the 20 typical meningiomas and both atypical meningiomas showed elevated choline. All the 22 cases demonstrated an alanine peak and another peak resonating at 3.8 ppm (possibly glutamate–glutathione complex). Lipid was elevated in four cases (three typical and one atypical meningiomas).

## Discussion

Several studies have attempted to differentiate meningioma grades and subtypes based on different MR parameters. However, none have compared the combination of DWI, DTI and perfusion findings with histopathology.

### Typical versus Atypical Meningiomas

The average size of meningiomas in this study was 5.0 cm ± 2.2 cm compared with Magill et al.’s report of 3.8 cm ± 1.8 cm.^9^ The mean size of atypical meningiomas (5.9 cm ± 0.7 cm) in this study was higher than that of typical meningiomas (4.6 cm ± 1.6 cm), and it was statistically significant (*p* = 0.038) with a cut-off value of 6.05 cm. One meningioma of the atypical type was 12.7 cm, which was considered as outlier for the statistical calculation of significance. This was histopathologically proven to be an anaplastic meningioma (World Health Organization [WHO] grade III), which has a propensity for rapid growth as pointed out before in literature.^9,10^

Ressel et al. also reported significantly larger sizes of atypical meningiomas compared to typical ones.^10^ Magill et al. identified the size of 3.2 cm as a cut-off point for the risk of being an atypical meningioma.^9^ The difference of the cut-off size in our study from that reported by Magill could be attributed to the limited number of cases. However, there is a difference in the mean size of the lesions of this study itself in comparison with that of Magill et al. This could be attributed to the timing of presentation and reflects the impact of socio-economic backgrounds on the results of various studies. Patients with milder symptoms may harbour lesions for a longer time and present with larger lesions, while patients reporting symptoms earlier may have smaller lesions. Similarly, lesions located near eloquent areas of brain will manifest symptoms early.

### Typical versus Atypical Meningiomas – Advanced MRI Parameters

The various advanced MRI parameters that were used for assessment in this study were ADC values, FA, MD, λ1, λ2, λ3, CL, CP, CS, rCBF, rCBV, MTT and CBF (ASL). The mean ADC values of typical and atypical meningiomas in previous studies and this study are listed in Table 4.

**TABLE 4:** A comparison between the mean and standard deviations of apparent diffusion coefficient values of typical and atypical meningiomas in previous studies and this study.

Study	Typical	Atypical
Lin et al. (3T)^11^	1.18†	1.03‡
Nagar et al. (1.5T)^12^	0.88 ± 0.08	0.66 ± 0.13
Hakyemez et al. (1.5T)^13^	1.17 ± 0.21	0.75 ± 0.21
**Gupta et al.** ^14^		
at 3T	0.82 ± 0.12	0.68 ± 0.10
at 1.5T	0.83 ± 0.11	0.70 ± 0.09
Filippi et al. (1.5T)^15^	1.03 ± 0.29	0.52 ± 0.12
Bano et al. (1.5T)^16^	1.04 ± 0.12	0.64 ± 0.05
**This study (3T)**	0.68 ± 0.11	0.57 ± 0.12

† , range: 1.08–1.30;

‡ , range: 0.398–1.15.

In all the mentioned studies, the mean ADC of atypical meningiomas is lower than that of typical meningiomas. This could be explained based on high cellularity, tumour matrices, fibrous or gliotic tissues, or a combination of these factors in atypical meningiomas. The ratio of intracellular to extracellular space determines the degree of water diffusion in biologic tissue and higher cellularity in atypical meningiomas may decrease the fraction of extracellular space, hence reducing net water diffusion.^12^ Increased mitotic activity, prominent nuclei, small cells with an increased amount of intracellular complex protein molecules, high nucleus-to-cytoplasm ratio and necrosis in atypical and malignant meningiomas contribute to reduced free diffusion of water.^17^ However, a statistically significant difference could not be established in our study, contrary to the previously documented statistical differences.^11,12,13,14,15,16^ This could be due to small number of atypical/malignant meningiomas in this study.

Moreover, the ADC values of meningiomas in this study was much lower than previous studies. One possible explanation could be that 9 of the 23 typical meningiomas were of the transitional subtype, which show lower ADC values and hence, the lower mean ADC.^18^ Another possible reason could be the higher *b*-value (2500 s/mm^2^) used in this study. All other previous studies were performed at *b*-values of 1000 s/mm^2^. Bano et al.^16^ also reported lower ADC values at a higher *b*-value (2000 s/mm^2^).

The mean FA values of typical and atypical meningiomas in previous studies and this study are listed in Table 5. The mean FA value of typical meningiomas in this study was in line with those of Toh et al.,^7^ Jolapara et al.^19^ and Aslan et al.^20^ The mean FA value of atypical meningiomas was similar to that of Aslan et al.^20^ However, the differences were not statistically significant.

**TABLE 5:** A comparison between the mean fractional anisotropy values of typical and atypical meningiomas in previous studies and this study.

Study	Typical	Atypical
Toh et al.^7^	0.230 ± 0.085 †	0.336 ± 0.105 †
Jolapara et al.^19^	0.285 ± 0.075 †	0.498 ± 0.04 †
Lin et al.^11^	0.54‡	0.59§
Aslan et al.^20^	0.21 ± 0.08 †	0.31 ± 0.13 †
Wang et al.^21^	-	-
**This study**	0.23 ± 0.08 †	0.26 ± 0.09 †

† , Lower fractional anisotropy values in atypical meningiomas. Actual values are not available in literature;

‡ , range: 0.41–0.83;

§ , range: 0.41–0.74.

Except for Wang et al.,^21^ all other studies (including this study) showed higher FA values in atypical meningiomas. Microscopic analysis of typical meningiomas has shown that they consist of oval or spindle-shaped neoplastic cells that form whorls, fascicles, cords or nodules. These microstructural elements in typical meningiomas act as physical barriers and stop water molecules from flowing linearly. On the other hand, atypical meningiomas show patternless or sheetlike growths. Hence, the water molecules travel more directionally in atypical meningiomas due to the absence of the physical barriers which are seen in typical meningiomas.^7,20^ The lack of a statistically significant difference between the two groups in this study may be due to the small number of cases in the atypical group. Another possible explanation may be due to differences in the number of various subtypes of typical meningiomas. Lower FA values in atypical meningiomas, as reported by Wang et al.,^21^ could be explained by the increased number of fibroblastic meningiomas in their study. Fibroblastic meningiomas have higher FA values,^22^ and hence, their mean FA value for typical meningiomas was higher relative to atypical meningiomas.

The lack of statistically significant differences in this study is in line with the previously reported study by Lin et al.^11^ However, Toh et al.^7^, Jolapara et al.^19^ and Aslan et al.^20^ reported significant differences in FA values between the two groups.

Few previous studies had tried to differentiate typical and atypical meningiomas based on perfusion (only rCBV). In this study, in addition to rCBV, we have also assessed rCBF and MTT for comparison. The means of perfusion parameters of typical and atypical meningiomas in previous studies and this study are listed in Table 6.

**TABLE 6:** A comparison between the mean perfusion parameters of typical and atypical meningiomas in previous studies and this study.

Study	Typical			Atypical		
	rCBF	rCBV	MTT	rCBF	rCBV	MTT
Zhang et al.^23^	-	7.16 ± 4.08	-	-	5.89 ± 3.86	-
Yang et al.^24^	-	8.02 ± 4.74	-	-	10.5 ± 2.1	-
**This study**	78.8 ± 72.4	13.4 ± 10.7	10.5 ± 2.8	66.4 ± 46.7	9.9 ± 6.7	9.9 ± 1.9

rCBF, relative cerebral blood flow; rCBV, relative cerebral blood volume; MTT, mean transit time.

In this study, we found higher rCBV values in typical meningiomas compared to atypical meningiomas. Similar results were found by Zhang et al.^23^ However, Yang et al. found completely contrasting results showing higher rCBV values in atypical meningiomas.^24^ The intravascular indicator dilution theory of Zierler forms the basis of the DSC perfusion MR imaging technique. It states that ‘in the absence of recirculation and contrast material leakage, CBV is proportional to the area under the contrast agent concentration–time curve’.^23^ Extravasation of contrast agent following administration of gadolinium leads to T1 effects, which might corrupt the evaluation of first-pass enhancement during perfusion imaging. A normal blood–brain barrier is fairly impermeable to gadolinium chelates because of the larger size of the molecule. As extra-axial lesions are present outside the blood-brain barrier, they are subjected to a substantial blood pool phase.^25^ Further, the tortuous and immature intratumoural vessels may increase the amount of contrast material that leaks from vessels into the extravascular space.^23^ This results in T1 effects due to the pooling of contrast material. Kimura et al. suggested: ‘Another problem associated with DSC perfusion is artificially lowered CBF values due to the delay and dispersion of tracer bolus during passage from the large vessel to brain parenchyma’.^26^ Hence, contrast perfusion techniques may not be accurate in extra-axial lesions.^25^

ASL uses a diffusible intrinsic tracer (electromagnetically labelled arterial blood water) to yield measurements of CBF, whereas DSC perfusion uses intravascular non-diffusible contrast media to yield measurements of CBV. Due to the constant exchange of water between tissue and capillary blood, ASL alters the net magnetisation in the tissue, which depends on the amount of perfusion.^26^ This could explain the discrepancy between the assessment of CBF by DSC perfusion and ASL. To the best of the authors’ knowledge, only two studies^26,27^ published in the English language have mentioned the use of ASL for comparing different grades of meningiomas. The mean CBFs of typical and atypical meningiomas were 161.09 ± 87.04 and 286.70 ± 8.06, respectively, and the difference between the two was statistically significant (*p* = 0.0000141). Receiver operator characteristic curve analysis suggested a cut-off value of 276.75 to differentiate between typical and atypical meningiomas with 66.7% sensitivity and 75.0% specificity. Higher CBF in atypical meningiomas could be explained based on their higher metabolic activity. Perfusion MRI detects the vascularity within a tissue and indirectly measures the tissue metabolic activity, since vasculature controls perfusion to fulfil metabolic demands of tissues.^28^

Of the 27 patients, MR spectroscopy yielded proper results in 22 patients. Twenty of these 22 cases, showed elevated choline and reduced NAA. This was similar to the results of Kinoshita et al.^29^ All the 22 cases revealed an alanine peak, which is considered the spectroscopic signature for meningiomas.^30,31,32^ Another distinct chemical compound resonating at 3.8 ppm peak was seen in each of the 22 cases. Kousi et al.^33^ reported that ‘this chemical substance observed at 3.8 ppm in short TE, might represent a Glx-a peak or Glx together with glutathione’. Tugnoli et al.^34^ identified in their *ex vivo* study that ‘the peak at 3.8 ppm receives contribution from phosphoethanolamine (PE) and amino acids, such as leucine, alanine, glutamate, glutamine, glutathione, lysine, arginine and serine’. Thus, in this study the peak at 3.8 ppm seen in all the cases could represent this glutamate–gluthathione complex.

No significant difference was found in the perilesional oedema associated with the two groups in this study. This suggests that brain oedema associated with meningiomas is possibly vasogenic in origin rather than due to tumour infiltration. However, Zikou et al.^28^ and Zhang et al.^23^ had found significantly higher mean rCBVs in the peritumoural oedema of atypical rather than benign meningiomas. These studies suggest a possibility of tumour infiltration into the perilesional oedema and/or higher peritumoural vascularity in higher grade meningiomas.

### Differentiating Between Subtypes of Typical Meningiomas

Among the 23 typical meningiomas, two were fibroblastic, nine were meningothelial and 9 were transitional type. Three were unspecified.

Most (seven out of nine) of the meningothelial meningiomas were homogeneous in appearance, which is the usual appearance.^17^ One of the fibroblastic meningiomas was hypointense on T2-weighted imaging, which is characteristic of fibroblastic meningiomas.^35^ Of the transitional meningiomas 77.8% (seven out of nine) showed heterogeneous post contrast enhancement, a classic feature of transitional meningioma.^35^

Though diffusion restriction has been considered a feature of atypical meningiomas,^35^ we found 88.9% (8 out of 9) transitional meningiomas showing diffusion restriction in this study. It was also seen in 66.7% (6 out of 9) meningothelial meningiomas. Hence, diffusion restriction may not be used as a criterion to differentiate typical and atypical meningiomas.

The mean ADC values of subtypes of typical meningiomas in a study by Hakyemez et al. were fibroblastic: 1.29 ± 0.28; meningothelial: 1.09 ± 0.20; transitional: 1.19 ± 0.07, whereas in this study, they were 0.76 ± 0.15, 0.73 ± 0.09 and 0.66 ± 0.07, respectively. In this study, lower ADC values were seen in transitional meningiomas as compared to meningothelial meningiomas. Transitional meningiomas have a combination of fibrous and meningothelial tissue.^35^ The fibrous component of transitional meningioma can reduce the fraction of extracellular space and hence result in diffusion restriction or reduced ADC values.

The mean FA values of subtypes of typical meningiomas in a previous study by Tropine et al.^22^ were fibroblastic: 0.396 ± 0.070; meningothelial: 0.196 ± 0.056; transitional: 0.296 ± 0.036, whereas in this study, they were 0.30 ± 0.02, 0.20 ± 0.07 and 0.22 ± 0.07, respectively. Mean FA value of fibroblastic meningiomas in a study by Jolapara et al. was 0.471 ± 0.03. All these studies showed higher FA values in fibroblastic meningiomas. This higher anisotropy can be credited to the regular cellular organisation within these tumours, thus allowing water molecules to travel more directionally. Whereas in meningothelial and transitional meningioma, the haphazard arrangement of cells contribute to isotropic diffusion and hence the relatively lower FA values.^7,22^

The mean rCBF and rCBV of fibroblastic meningiomas in this study were significantly lower than those of meningothelial meningiomas (*p* < 0.05). This is similar to the results of Zhang et al.^36^ and Kimura et al.^26^ This suggests that, among the subtypes of typical meningiomas, fibroblastic meningiomas have the lowest vascularity.

In this study, the mean rCBV and CBF (ASL) values of perilesional oedema associated with transitional meningiomas were significantly higher than those associated with meningothelial meningiomas. Also, a higher incidence of perilesional oedema was seen with transitional meningioma. It was also observed that extensive brain oedema was associated with typical meningiomas with higher CBFs, most of which were transitional meningioma. Based on this, a possible correlation between CBF and perilesional oedema could be suggested.

Surgical excision is the treatment for meningiomas. Knowing the consistency of the tumour preoperatively can help in planning the surgery better.^1^ Consistency of meningioma depends on tumour cellularity, water content and fibrous content, which in turn depends on the meningioma subtype.^37,38^ Hyperintensity on T2WI correlates well with soft tumours, which may be attributed to higher water content and increased vascularity while the lower signal on T2WI for hard tumours might be due to less water, higher cellularity and more collagen and calcium.^1,37,38^ Hard lesions show lower ADC values and higher FA values. Fibroblastic meningiomas are typically hard in consistency and appear hypointense on T2WI.^37^ They are associated with increased risk of postoperative cranial nerve deficits.^38^ Chromosome 22q abnormalities are also more common in transitional and fibrous meningiomas.^28^

Meningothelial, angioblastic or atypical meningiomas are hyperintense on T2WI.^38^ The meningothelial variant is associated with an activating E17K mutation in the AKT1 gene, which can act as therapeutic targets of AKT inhibitors, thus holding prognostic and future therapeutic implications.^28^ In case of a high-grade atypical meningioma, alterations in the planned size, shape and extent of a craniotomy can ensure clean margins by achieving complete resection.^24^ Hence, preoperative diagnosis of meningioma grade and subtype can help in better risk–benefit assessment, predict length of operating time, expectations regarding the extent of resection and the likelihood of need for adjuvant therapy, all of which can be very helpful in patient counselling regarding surgical outcome.

### Limitations of the Study

The patients included were by purposive sampling in a hospital setting. Therefore, the data are limited to patients with impairing/disabling symptoms and might not be totally representative of the findings in the patients with early stage lesions who do not report to the hospital. Moreover, the sample size of 27 is small for an accurate depiction of such a wide variety of meningiomas and larger studies are needed for more definitive results. The number of high-grade meningiomas was very small, which may render these findings as less conclusive. Less common subtypes of WHO grade I meningiomas such as angiomatous, microcystic, secretory and metaplastic meningiomas and other WHO grade II meningiomas (i.e. chordoid and clear cell meningiomas) were not seen during this study period.

## Conclusion

Advanced MRI brain sequences proved to be useful in differentiating typical and atypical/anaplastic meningiomas as well as in the subtyping of typical meningiomas. The tumour size and ASL perfusion were the two parameters, which could differentiate between typical and atypical meningiomas. Atypical meningiomas have higher size and CBF values as compared to typical meningiomas. ADC, FA, rCBF and rCBV are crucial in distinguishing different subtypes of typical meningiomas. Transitional meningiomas have lower ADC values while fibroblastic meningiomas have higher FA values and lower vascularity (rCBF and rCBV). Considering the wide variety of meningiomas, studies with a larger sample size can be conducted to elucidate their individual characteristics further. Since, the incidence of higher-grade meningiomas is less frequent, multicentric studies may be conducted to collate an adequate sample of higher-grade meningiomas for a more definitive conclusion. Identifying the grade and subtype non-invasively can help in proper planning of treatment and improve clinical outcomes.
